# Percutaneous Thrombectomy for the Treatment of Lower Extremity Deep Vein Thrombosis: Medium-Term Follow-up Results and Analysis of 112 Cases

**DOI:** 10.7759/cureus.22689

**Published:** 2022-02-28

**Authors:** Hakki Kursat Cetin, Emrah Sevgili

**Affiliations:** 1 Cardiac Surgery, Sisli Etfal Training and Research Hospital, Istanbul, TUR; 2 Cardiology, Avcilar Hospital, Istanbul, TUR

**Keywords:** success rate, percutaneous thrombectomy, lower extremity, follow-up, deep vein thrombosis

## Abstract

Introduction

The aim of this study was to analyze percutaneous thrombectomy (PT) outcomes for the management of lower extremity deep vein thrombosis (DVT) with medium-term follow-up.

Methods

The study included charts of patients who underwent PT due to lower extremity DVT between August 2017 and March 2021. Patient characteristics and procedure outcomes were recorded in the electronic information system on the same day as the procedure. The procedures with complete removal of thrombus following PT without requiring additional procedure or additional thrombectomy apparatus were considered successful. Also, duration of follow-up was noted.

Results

In total, 112 patients were enrolled in the study. The femoropopliteal (40.2%) and iliofemoral (25.0%) veins were the most common sites with thrombus detected. The duration of PT procedure and fluoroscopy were 123.1 minutes and 21.9 minutes, respectively. Estimated blood loss was 255.1 milliliters. The hospital stay and intensive care unit stay following PT were 3.7 and 1.4 days, respectively. Major hemorrhage did not occur in any patient, but we encountered bradycardia in six (5.4%) patients, acute renal failure in one (0.9%) patient, hemoglobinuria in 11 (9.8%) patients, leg pain in 15 (13.4%) patients, and pulmonary embolism in 2 (1.8) patients, respectively. Success of the present study was 94.6% in the first month, and re-operation was required only in one patient. The mean follow-up period was 21.1 months with 90.2% venous patency rates.

Conclusion

The present study demonstrated that PT was an effective and reliable treatment modality with acceptable complication rates for the treatment of lower extremity DVT. Additionally, the efficacy of PT was proven by mid-term follow-up results.

## Introduction

Deep vein thrombosis (DVT) is defined as abnormal clot formation in veins and lower extremity veins are the most common sites of DVT [1]. Approximately, one per 1,000 individuals is faced with DVT and DVT-related complications annually, and the risk of DVT increases with age, obesity, immobilization, and presence of malignant tumors. Pain, swelling, venous ulceration, and worsening quality of life are common conditions in patients who experience DVT. In addition, extremity amputations and pulmonary thromboembolism can be seen in patients with untreated DVT [2]. Long-term anticoagulation therapy from three to six months is the most widely accepted treatment modality for DVT. Nevertheless, long-term anticoagulation administration includes possibilities of irregular anticoagulation use, interactions between anticoagulants and other medications, and life-threatening bleeding [3]. Due to the aforementioned risks, fast-acting treatment options with high success and low complication rates are being investigated for patients with DVT.

Technological developments in medicine presented new treatment modalities for DVT, such as catheter-assisted thrombolysis, intravenous thrombolysis, and percutaneous thrombectomy (PT). Benarroch-Gampel et al. performed PT for lower extremity DVT in 12 patients and achieved complete removal of thrombus and symptom resolution in 11 patients [4]. In another study by Dumantepe and Uyar, PT was performed for lower extremity DVT, and the authors obtained a success rate of 85% following the procedure without any significant complications [5].

Although previous studies investigated the role of PT in the treatment of lower extremity DVT, these studies have conflicting results and small patient numbers without follow-up results. To our knowledge, the present study includes the largest case series to analyze PT outcomes for the management of lower extremity DVT with medium-term follow-up.

## Materials and methods

The study included charts of patients who underwent PT due to lower extremity DVT (popliteal, femoral, external iliac, and common iliac veins) between August 2017 and March 2021. Operation technique and possible outcomes of PT were explained in detail to all patients, and informed consent was obtained 24 hours before the procedure. The study was conducted in accordance with the Declaration of Helsinki. Patient characteristics and procedure outcomes were recorded in the electronic information system on the same day as the procedure. Additionally, all patient data and findings were recorded instantly during follow-up. Diagnosis of DVT was made by physical examination and lower extremity venous duplex ultrasound. Also, compressibility, echogenicity and extent of thrombus, and procedure success were determined by venous duplex ultrasound. The presence of bilateral lower extremity DVT, active skin infection at the percutaneous access site, and untreated psychiatric disorder were exclusion criteria in the present study. In addition, patients under the age of 18 years were excluded from the study.

Patient characteristics including age, body mass index (BMI), gender, smoking habit or not, presence of hypertension and diabetes mellitus, and coexistent malignancy were recorded. Also, DVT history, patient symptoms associated with DVT, duration of symptoms, anatomical location of thrombus and side of DVT, and lesion length were noted. Procedure-related parameters including operation time, fluoroscopy time, amount of blood loss, and number of implanted stents were registered in the electronic data system. Lastly, duration of hospital and intensive care unit (ICU) stay, decrease in hemoglobin level, re-operation rate, complications, and success were recorded. The procedures with complete removal of thrombus following PT without requiring additional procedure or additional thrombectomy apparatus were considered successful. Also, the duration of follow-up was noted.

Procedure technique

All PT procedures were performed in the same manner. In an angiography lab with fluoroscopy, local anesthesia and conscious sedation were administered to all patients, and 5,000 IU of heparin sodium was administered preoperatively. In addition, heparin sodium was given to maintain therapeutic levels during the procedure. To prevent pulmonary embolism, venous access was obtained from the contralateral femoral vein, a 0.035-inch guidewire was advanced to the vena cava, and a vena cava filter was placed just below the renal veins. After sterilization of the access area, an 18-gauge needle was used for popliteal venous puncture under ultrasonography assistance and an introducer sheath with 8 F size was inserted. Venography was performed to evaluate the location and extension of thrombus, and the guidewire was advanced through the thrombus. The PT procedure was performed in a repetitive pattern until complete removal of the venous thrombus. After the entire thrombus was removed, phlebography was performed to assess venous patency and additional procedure requirements. Compression stockings were placed on the affected extremity. Success of PT was evaluated one month after the procedure with venous duplex ultrasound, and all patients were re-evaluated in the 6th, 12th, 18th, 24th, 30th, and 36th months after the procedure.

Statistical analysis was performed with the Statistical Package for the Social Sciences Version 25 (SPSS, IBM Corp., Armonk, NY, USA). The data were reported in a descriptive fashion including frequency, mean, and standard deviation. The Kaplan-Meier curve was applied to venous patency times.

## Results

In total, 112 patients who underwent PT for lower extremity DVT were enrolled in the study, and five patients were excluded from the evaluation due to study exclusion criteria (three patients had bilateral lower extremity DVT, one patient had a psychiatric disease, and one patient was ≤18 years). The mean age was 54.6 years in the study population, and 55.4% of patients were male. The mean BMI was 28.2 kg/m^2^, and 61 (54.5%) patients were smokers. The presence of diabetes mellitus, hypertension, and malignant tumor were detected in 18 (16.1%), 35 (31.2%), and 60 (53.6%) patients, respectively. The most common symptoms were pain (108 of 112 patients) and swelling (110 of 112 patients). The mean duration of symptoms was 6.7 days. Pre-procedural patient characteristics are summarized in Table 1.

**Table 1:** Preoperative demographic data of patients *Mean ± standard deviation ASA, American Society of Anesthesiologists Classification; DVT, deep vein thrombosis; PE, pulmonary embolism

	n=112
Age (years)*	54.6±16.5
Gender	
Male	62 (55.4%)
Female	50 (44.6%)
BMI (kg/m^2^)*	28.2±4.0
ASA score*	1.9±0.5
Smoking status	61 (54.5%)
Diabetes mellitus	18 (16.1%)
Hypertension	35 (31.2%)
Coexistent malignancy	60 (53.6%)
DVT history	57 (50.9%)
Symptoms	
Swelling	110 (98.2%)
Pain	108 (96.4%)
Additional PE	10 (8.9%)
Duration of symptoms (days)*	6.7±3.9

The femoropopliteal (40.2%) and iliofemoral (25.0%) veins were the most common sites with thrombus detected. The mean lesion length was calculated was 11.0 cm. The duration of PT procedure and fluoroscopy were 123.1 minutes and 21.9 minutes, respectively. Estimated blood loss was 255.1 milliliters and stenting rate was 3.6% (four of 112 patients) (Table 2).

**Table 2:** Operative data of patients *Mean ± standard deviation DVT, deep vein thrombosis

	n=112
Site of DVT	
Iliofemoral	28 (25.0%)
Popliteal	22 (19.6%)
Femoral	17 (15.2%)
Femoral/popliteal	45 (40.2%)
Side involved	
Right	57 (50.9%)
Left	55 (49.1%)
Lesion length (cm)*	11.0±1.9
Operation time (min)*	123.1±25.9
Flouroscopy time (min)*	21.9±10.5
Amount of blood loss (mL)*	255.1±45.2
Stenting rate	4 (3.6%)
No. of implanted stent*	1.6±1.1

**Figure 1:**
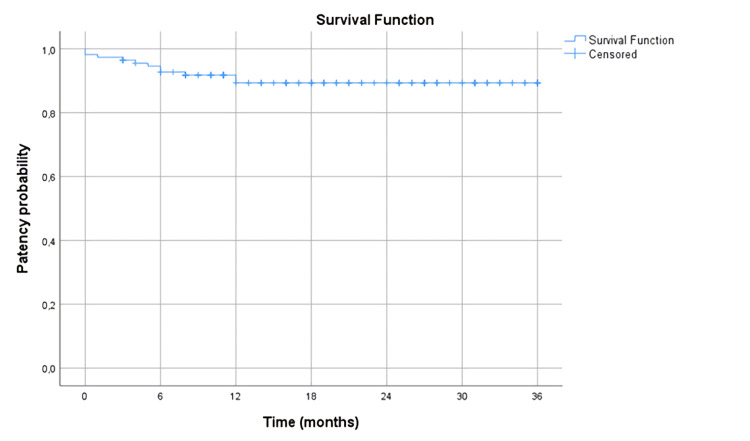
Survival analysis of freedom from loss of patency demonstration Kaplan–Meier probability estimates.

The hospital stay and ICU stay following PT were 3.7 and 1.4 days, respectively. The mean drop in hemoglobin level was 1.2 g/dL. Major hemorrhage did not occur in any patient, but we encountered bradycardia in six (5.4%) patients, acute renal failure in one (0.9%) patient, hemoglobinuria in 11 (9.8%) patients, leg pain in 15 (13.4%) patients, and pulmonary embolism in two (1.8) patients, respectively. Additionally, no patient died due to the procedure or procedure-related complications. Success of the present study was 94.6% in the first month, and re-operation was required only in one patient (Table 3). The mean follow-up period was 21.1 months, and venous patency rates are summarized in Figure 1.

**Table 3:** Postoperative outcomes and complications *Mean ± standard deviation ICU, intensive care unit

	n=112
Hospital stay (days)*	3.7±1.5
ICU stay (days)*	1.4±1.0
Success (after procedure)	106 (94.6%)
Success (at an average follow-up of 21.1 months)	101 (90.2%)
Decrease in hemoglobin (g/dlL*	1.2±1.0
Complications	
Bradycardia	6 (5.4%)
Acute renal failure	1 (0.9%)
Hemoglobinuria	11 (9.8%)
Leg pain	15 (13.4%)
Major hemorrhage	0 (0%)
Pulmonary embolism	2 (1.8%)
Re-operation	1 (0.9%)
Mortality	0 (0%)
Follow-up (months)*	21.1±8.9

## Discussion

Previous reports stated that after a new surgical technique is developed, it is important to demonstrate the effectiveness and reliability of the technique in large case series [6]. PT is a relatively new alternative to anticoagulant therapy for the management of patients with lower extremity DVT. However, previously published articles on the efficiency and safety of PT were either case reports or case series involving a small number of patients. To our knowledge, the present study includes the largest number of patients evaluating the effectiveness of PT for lower extremity DVT. We achieved success rates of 94.6% and 90.2%, respectively, after the procedure and at an average follow-up of 21.1 months with acceptable complication rates.

The main goals of PT in the treatment of lower extremity DVT are complete removal of thrombus, recanalization of vein, and improvement of patient symptoms [7]. Previous studies that investigated the success of PT for lower extremity DVT had limited numbers of patients. Ozpak et al. investigated the efficiency of PT for lower extremity DVT in only 21 patients and achieved success rates of 95% and 85% in the first and sixth months after the procedure, respectively [8]. In another study, Loffroy et al. analyzed the success of PT in 30 patients with acute lower extremity DVT, and the authors removed thrombus successfully in all patients. However, Loffroy et al. encountered early thrombosis in three patients (10% of study population) [9]. We achieved a re-canalization rate of 94.6% in the present study.

While many studies focused on early success after PT, most of them do not include medium- and long-term results for various reasons. Demirtürk et al. stated that success of PT in the management of iliofemoral DVT decreased from 82% in the first year to 58% in the fifth year. Although the mean follow-up period was 30 months, Demirtürk et al.’s study included only 18 patients [10]. In another study, Kim et al. evaluated the 32.1-month follow-up results of PT for lower extremity DVT treatment, and recurrent DVT was detected in two of 19 patients. However, the study population included a relatively small number of patients, and six patients were lost during follow-up in Kim’s study (31.6% of study population) [11]. The present study included the medium-term results of 112 patients, and we achieved a success rate of 90.2% in the 21.1-month follow-up period.

Complications are the most undesirable events of invasive procedures [12]. Dumantepe and Uyar stated that slight leg pain and hemoglobinuria were the most common complications after PT with 16.1% and 11.7% rates, respectively [5]. Demirtürk et al. reported three complications including venous dissection in two patients and in-stent thrombosis in one patient; however, the author did not report post-procedure complications [10]. Wang et al. found that most hemorrhagic complications were mild following PT and that the incidence of major bleeding was 4.6%. Additionally, Wand et al. claimed that cases with anemia requiring blood transfusion increased that rate [13]. In our study, most of the complications were acceptable (leg pain in 13.4% of patients and hemoglobinuria in 9.8% of patients), although we faced more serious complications including acute renal failure in one patient and pulmonary embolism in two patients.

Reflecting the experience of a single center can be considered as a limitation of the study. Secondly, we did not analyze the effect of learning curve on PT success and complications. We believe that the effect of the learning curve on PT for the treatment of lower extremity DVT could be the subject of another study. Additionally, this study did not focus on the cost of PT procedures, which could be a target for a different study. Also, we did not keep records of the number of punctures while obtaining venous access and number of maneuvers for complete thrombus removal. Lastly, patient quality of life and post-procedure pain levels were not analyzed in the present study, which may be subjects of further studies.

## Conclusions

The present study demonstrated that PT was an effective and reliable treatment modality with acceptable complication rates for the treatment of lower extremity DVT. Also decrease in hemoglobin level, hospitalization time, and re-operation rate were acceptable following PT. Additionally, efficacy of PT was proven by mid-term follow-up results. Our study findings must be supported by further prospective studies including larger patient numbers and data from more than one center.
